# Hybrid Data–Mechanistic Approach to Sensor Fault Detection for Direct-Drive Electric Vehicle Motors

**DOI:** 10.3390/s26196111

**Published:** 2026-09-26

**Authors:** Min Wang, Xiaoyu Wang, Te Chen, Zaijuan Li, Fei Tian

**Affiliations:** 1 School of Mechanical and Electrical Engineering, Suzhou Polytechnic University, Suzhou 215000, China; 01208@jsspu.edu.cn (M.W.); 92008@jsspu.edu.cn (Z.L.); 01123@jsspu.edu.cn (F.T.); 2 Robotics and Intelligent Equipment Engineering Research Center of Jiangsu Province, Suzhou Polytechnic University, Suzhou 215000, China; 3 Automotive Engineering Research Institute, Jiangsu University, Zhenjiang 212013, China; 1000005564@ujs.edu.cn

**Keywords:** electric vehicles, direct-drive motor, fault diagnosis, residual filtering, signal interference

## Abstract

Sensor fault diagnosis for direct-drive electric vehicle in-wheel motor systems faces significant challenges under complex operating conditions. Specifically, signal disturbances such as noise interference, model uncertainty, and environmental factors often cause residual distortion, masking fault features and increasing false alarm rates. To address these challenges, a fault diagnosis method based on data-driven subspace identification and residual anti-interference filtering is proposed. Firstly, a state space model of the permanent magnet brushless DC motor is established, and a subspace identification algorithm is combined to construct a discrete system model considering disturbances. An adaptive residual filter is designed to suppress the influence of disturbances on residual signals, and predictive time-domain rolling optimization of residual estimation is introduced. Furthermore, the maximum likelihood ratio is used to construct adaptive thresholds to enhance fault sensitivity and robustness. The bench and real vehicle experiments show that this method can effectively extract fault features under noise and uncertainty interference, significantly improve the fault localization accuracy of current and speed sensors, reduce false alarm rates, and provide technical support for the reliable operation of direct-drive motor systems.

## 1. Introduction

Many key systems of electric vehicles rely on sensors to monitor and control their operating status. Once a sensor malfunctions, it may lead to serious problems, such as abnormal vehicle power system operation, drive and brake failures, and even vehicle safety issues [1,2,3]. Through effective fault diagnosis techniques, sensor faults can be identified and located in a timely manner, and corresponding measures can be taken to avoid serious accidents caused by sensor failures [4,5,6].

Electric vehicles are complex electromechanical integrated systems, where various components work together and are closely connected [7,8,9]. As an important component for vehicles to perceive the external environment and their own state, the failure of sensors may trigger a series of chain reactions, leading to the failure or performance degradation of other systems. For the direct-drive wheel-hub motors of distributed-drive electric vehicles, the current and speed sensors in the motor controller are important for monitoring motor operating status [10,11,12]. If the corresponding sensor malfunctions, it may affect the speed control of the motor, which may lead to abnormal power output of the vehicle or affect the execution of the vehicle chassis dynamics control system. Accurate sensor function is crucial for the efficient operation and active safety of the vehicle drive system [13,14,15].

Generally, the process of fault diagnosis consists of several sequential aspects: fault detection, fault isolation or localization, fault magnitude estimation, and in certain cases, fault classification [4,5,6]. For direct-drive electric vehicles, timely fault detection and accurate localization of sensor faults are of paramount importance to ensure vehicle safety and prevent cascading failures. While fault estimation and classification are also valuable research directions, this study primarily focuses on the fault detection and localization aspects for current and speed sensor gain faults. Specifically, the proposed method aims to rapidly detect the occurrence of a fault and accurately isolate the faulty sensor under complex disturbed environments, either the current sensor or the speed sensor, without delving into precise magnitude estimation or multi-type classification.

Effective sensor fault diagnosis typically relies on residual signals generated by comparing sensor measurements with model-based state estimates. Consequently, accurate model identification is fundamental to precise fault diagnosis. However, in the context of the proposed hybrid data–mechanistic approach, unavoidable signal disturbances (e.g., noise and model uncertainty) can directly affect the identification accuracy of the motor model [16,17,18,19], leading to biased state estimates and distorted residuals that severely compromise fault detection and localization. In data signals, disturbance factors can interfere with the accuracy and reliability of the original signal. From the perspective of model identification, models are built on data to describe the behavior and characteristics of a system. When the data signal is disturbed, these disturbed signals serve as the basis for model identification, which can cause bias in the estimation of model parameters [20,21,22]. Disturbance factors may alter the distribution characteristics of data signals, such as mean and variance. Model identification usually assumes that the data follows a certain distribution pattern. When disturbances cause changes in the data distribution, the parameter estimation method based on the original assumption will fail. Moreover, disturbances may introduce outliers or noise, which can interfere with the model’s exploration of intrinsic relationships in the data, thereby affecting the accuracy of model identification [23,24,25,26]. Therefore, it is crucial for sensor fault diagnosis to address the impact of signal disturbances on the accuracy of motor model identification, in order to obtain more accurate and reliable model state values and residual values [27,28,29].

Residual-based fault diagnosis is one of the most widely used model-based approaches, where a residual signal is generated by comparing the sensor measurements with the outputs predicted by the system model. Under the assumption of an accurate model and disturbance-free conditions, the residual remains near zero in fault-free scenarios; once a sensor fault occurs, the residual deviates from zero, thereby enabling fault detection. The impact of interference on residual signals and the effectiveness of fault diagnosis cannot be ignored. Accurate fault diagnosis relies on residual signals that can truly reflect the operating status and fault characteristics of the system [30,31,32]. When interference signals are mixed with fault characteristic signals, the residual signal becomes complex and fuzzy, making it difficult to accurately determine the type, location, and severity of the fault, thereby reducing the accuracy of fault diagnosis [33,34,35]. Due to the presence of interference, residual signals may frequently exceed a predetermined fixed threshold, resulting in a large amount of false-alarm fault information. In addition, in some cases, interference may also mask residual changes caused by faults, resulting in faults not being detected in a timely manner, leading to missed reports and affecting the safe operation of the motor system and even the entire vehicle. Therefore, in this case, in addition to filtering the residual signal, the reasonable setting of adaptive thresholds is also of great significance for improving the effectiveness of fault diagnosis [36,37,38]. To distinguish fault-induced residual deviations from disturbance-induced fluctuations, a predefined threshold is typically employed for fault decision-making. Fixed thresholds, however, struggle to adapt to varying disturbance intensities. In contrast, adaptive thresholds can dynamically adjust according to the statistical characteristics of disturbances, and various works have modeled these adaptive thresholds based on methods such as moving averages, robust estimation, Kalman filtering, and generalized likelihood ratios (GLRs). Nevertheless, few studies have jointly considered disturbance-aware model identification, residual filtering, and adaptive threshold design within a unified framework.

Generally, existing fault diagnosis methods have evolved along three main pathways: model-based, purely data-driven, and, bridging the two, a hybrid physics-informed data–mechanistic approach [39,40,41]. Model-based methods rely on accurate physical models but are sensitive to model mismatch and external disturbances, whereas purely data-driven methods circumvent model dependency at the cost of requiring abundant historical fault data and often lacking physical interpretability. These complementary limitations have motivated a growing body of work on hybrid approaches that synergistically combine physical models with data-driven techniques. Although significant progress has been made in sensor fault diagnosis for motor systems, existing methods still share the following common deficiencies when confronted with signal disturbances [42,43]. First, model-based methods exhibit certain robustness to specific types of uncertainties. However, in practical applications, the system often operates under mixed-disturbance environments, where model uncertainty and measurement noise are coupled. Under such coupled disturbances, their residual signals are prone to distortion, masking fault features or generating excessive false alarms [44,45,46]. Second, pure data-driven methods circumvent the need for accurate models, yet they typically lack explicit mechanisms to mitigate the impact of disturbances on residual signals, leading to significantly degraded identification accuracy under strong noise and operating condition variations. Consequently, under mixed-disturbance environments where model uncertainty and measurement noise are coupled, their residual signals are prone to distortion, masking fault features or generating excessive false alarms. Third, most existing methods employ fixed thresholds for fault decision-making, which cannot adapt to dynamically varying disturbance intensities, making it difficult to reconcile detection sensitivity with false alarm rates [47,48].

It is worth noting that the focus of sensor fault diagnosis has recently progressed towards fault reconstruction, which aims to estimate the exact profile of faults rather than merely detecting their occurrence [49,50,51]. Reviewing the state of the art in sensor fault diagnosis for motor systems, existing methods mainly evolve along three technical trajectories: Model-based approaches [2,17,18,19,30,33] rely on precise mechanistic descriptions and exhibit robustness against specific uncertainties, but residual signals are prone to distortion under the coupled noise and model uncertainty in direct-drive in-wheel motors, masking fault features. Data-driven subspace identification [9,14,15,20,27,31] circumvents the need for precise mechanistic models, yet existing studies mostly focus on single-loop optimization of identification accuracy or residual generation, lacking active disturbance suppression mechanisms. Identification accuracy degrades significantly when strong noise couples with time-varying operating conditions, and unfiltered residuals have limited capability to characterize fault features. Lastly, statistical inference-based adaptive threshold methods [12,16,25,32,34,52,53] such as the generalized likelihood ratio test can theoretically balance detection sensitivity and false alarm rate by dynamically matching disturbance intensity via chi-square distribution, but the performance of these methods depends greatly on the purity of upstream residuals, which is constrained by the quality of preceding modules. Current research mostly adopts single-loop optimization or pairwise combinations, with limited work targeting the complex multi-disturbance coupled conditions of direct-drive in-wheel motors. In particular, few studies integrate disturbance-aware predictive-horizon subspace identification, SVD-optimized residual anti-interference filtering, and adaptive threshold decision-making within a collaborative, unified framework.

Notably, the aforementioned three trajectories mostly appear in the existing literature as single-loop optimization or pairwise combinations: model-based observer methods are often paired with filtering techniques; data-driven subspace identification is frequently combined with fixed thresholds or simple exponentially weighted moving average filters; and adaptive threshold methods mostly serve single model architectures. There is limited research addressing the complex disturbance environment of direct-drive in-wheel motors, in which noise interference, model uncertainty, and time-varying operating conditions are coupled. Few studies integrate disturbance-aware predictive-horizon subspace identification, SVD-optimized residual anti-interference filtering, and maximum likelihood ratio-based adaptive threshold decision-making within a unified framework for collaborative design and joint optimization.

To address this gap, this paper proposes a sensor fault diagnosis method that integrates data-driven subspace identification with residual anti-interference filtering. Specifically, this hybrid approach is designed to simultaneously tackle the aforementioned deficiencies: it combines mechanistic models with data-driven subspace identification to mitigate disturbance-induced model inaccuracies, introduces an SVD-optimized residual filter to provide explicit disturbance suppression on residual signals, and employs a maximum likelihood ratio-based adaptive threshold to dynamically reconcile detection sensitivity with false alarm rates.

The core contribution lies in firstly constructing a framework that combines mechanistic models with data-driven subspace identification, effectively addressing the impact of disturbances. The residual anti-interference filter based on singular value decomposition optimization significantly suppresses the interference of disturbances on residual signals, greatly improving the authenticity and robustness of fault characterization. Secondly, using an adaptive threshold based on a maximum likelihood ratio instead of a fixed threshold can dynamically adapt to complex disturbance environments and effectively reduce the false alarm rate in fault detection. The effectiveness of the method was fully verified through bench and real vehicle experiments. Under strong noise and model uncertainty interference, the state identification errors of current and speed were significantly reduced: when a 5 times gain fault was injected into the current or speed sensor, the system accurately diagnosed and identified the fault after it occurs. The GLR value of the fault signal significantly exceeded the set threshold, achieving rapid diagnosis and accurate positioning of highly sensitive and high-precision faults. This method provides a highly robust online sensor fault diagnosis solution for direct-drive electric vehicles, especially in complex driving conditions that involve temperature changes and vibration, and has important engineering application value.

The rest of this paper is organized as follows. The mathematical model of the direct-drive in-wheel motor system is described in Section 2. The residual generation and fault diagnosis of sensor faults in the direct-drive motor system are presented in Section 3. The experimental verification of a bench test and actual vehicle is provided in Section 4, and the concluding remarks are presented in Section 5.

## 2. Mechanistic and Data-Driven Models of the Direct-Drive In-Wheel Motor System

The mechanistic model [9,19] clarifies the causal relationship of variables based on physical laws, while the data-driven model explores complex patterns in actual data. The combination of the two enables the hybrid model to reflect the physical characteristics of the system and correct errors caused by simplifying assumptions, thus more accurately describing the dynamic characteristics of the system and adapting to different working and load conditions. In addition, the mechanistic model provides the basis for the system’s physical structure and main dynamic characteristics, while the data-driven model further optimizes and supplements it. This approach reduces the difficulty and workload of system identification and improves identification efficiency.

The torque balance equation for the output shaft of a permanent magnet brushless DC motor [18,33] is

(1) $$J\dot{\omega }+b\omega =K_{t}i-T_{l},$$

The dynamic voltage balance equation of the equivalent circuit of a permanent magnet brushless DC motor is

(2) $$U=Ri+L\dot{i}+K_{a}\omega ,$$

where *J* is the moment of inertia of the motor rotor, *b* is the damping coefficient, *K*_t_ is the motor torque constant, *T_l_* is the motor load torque, *i* is the line current, *U* is the line voltage, ω is the motor speed, *R* is the equivalent line resistance of the winding, *L* is the equivalent inductance of the winding, and *K_a_* is the back electromotive force coefficient.

If the state vector *x* contains the motor current and speed, and the input *u* contains the motor voltage *U* and load torque *T_L_*, and *y* is the sensor measurement output, then the state space equation of the permanent magnet brushless DC motor model can be expressed as

(3) $$\dot{x}=Ax+Bu,$$

(4) y=Cx,

where $x=\left[\begin{array}{c} i\\ \omega \end{array}\right]$, $A=\left[\begin{array}{cc} -\frac{R}{L} & -\frac{K_{a}}{L}\\ \frac{K_{t}}{J} & -\frac{b}{J} \end{array}\right]$, $B=\left[\begin{array}{cc} \frac{1}{L} & 0\\ 0 & -\frac{1}{J} \end{array}\right]$, $u=\left[\begin{array}{c} U\\ T_{l} \end{array}\right]$, $C=\left[\begin{array}{cc} 1 & 0\\ 0 & 1 \end{array}\right]$.

To establish a high-precision identification model of the permanent magnet brushless DC motor, a comprehensive test system was designed and built to obtain the multi-dimensional dynamic data during the operation of the motor. In this work, “hybrid data–mechanistic” specifically refers to the combination of the physics-based mechanistic model in Equations (1)–(4) and the data-driven subspace identification (N4SID) for accurate motor modeling under disturbances. The experimental platform adopts a three-axis linkage structure, and the driving motor, dynamic-parameter detection unit and load simulation device are connected coaxially through a high-precision fixture. To ensure the coaxial accuracy of mechanical transmission, each component base is equipped with a horizontal calibration mechanism. The power supply system uses a DC voltage stabilizing mode, while a magnetic powder brake is used to simulate the dynamic load torque.

To solve the possible influence of rising motor temperature on the stability of the test data, the system applies a specially designed dual-mode temperature control scheme. On the one hand, the high-power axial fan is used to implement forced air cooling for the main body of the motor; on the other hand, the magnetic powder brake is equipped with an independent closed-loop water-cooling circulating device. The control system adopts a layered signal input structure, and the program-controlled signal generator generates a PWM modulation waveform to drive the motor controller, and accurately adjusts the duty cycle to simulate the power demand under different working conditions.

The data acquisition system integrates a module for synchronous detection of multiple physical quantities. The current and voltage sensors based on the Hall principle are responsible for real-time monitoring of armature loop parameters, while the dynamic torque and speed sensors continuously capture the speed torque characteristic curves under different working conditions. The step-loading strategy is adopted in the test process, and the command voltage of the motor controller is gradually increased with 0.2 V as the step value to ensure the complete coverage of the entire working range of the motor. The sample dataset is obtained through multiple groups of working condition tests, and the data partition storage mechanism is adopted to divide the collected data sample into model identification and verification sets, which are used for data-driven model training and subsequent model verification.

Figure 1 shows the dynamic response curve of the motor’s state parameters under the bench-test conditions, including bus voltage, line current, output torque, and speed. The dataset clearly captures the underlying characteristics of the motor’s electromechanical energy conversion process and the coupled variations in multiple parameters.

## 3. Residual Generation and Fault Diagnosis of Sensor Faults in the Direct-Drive Motor System

### 3.1. Subspace Identification Algorithm for the Motor Model Considering Signal Disturbance

The motor model in Equations (3) and (4) can be represented as a discrete linear time invariant system in the following form

(5) $$\left\{\begin{array}{l} x(k+1)=Ax(k)+Bu(k)+Ke(k)\\ y(k)=Cx(k)+Du(k)+e(k) \end{array}\right.,$$

where *x(k)* is the system model state value, *u(k)* is the system model input value, *e(k)* is the innovation sequence, *K* is the filtering gain, and *y(k)* is the system model measurement value.

For the discrete identification model of the motor in Equation (5), the Hankel matrices for its input and output are represented as

(6) $$U_{0\left|i-1\right.}\overset{def}{=}\left(\begin{array}{cccc} u_{0} & u_{1} & \ldots & u_{j-1}\\ u_{1} & u_{2} & \ldots & u_{j}\\ \vdots & \vdots & \vdots & \vdots \\ u_{i-1} & u_{i} & \ldots & u_{i+j-2} \end{array}\right),$$

(7) $$Y_{0\left|i-1\right.}\overset{def}{=}\left(\begin{array}{cccc} y_{0} & y_{1} & \ldots & y_{j-1}\\ y_{1} & y_{2} & \ldots & u_{j}\\ \vdots & \vdots & \vdots & \vdots \\ y_{i-1} & y_{i} & \ldots & u_{i+j-2} \end{array}\right),$$

where $U_{p}\overset{def}{=}U_{0\left|i-1\right.}$, $U_{f}\overset{def}{=}U_{i\left|2i-1\right.}$, $Y_{p}\overset{def}{=}Y_{0\left|i-1\right.}$, $Y_{f}\overset{def}{=}Y_{i\left|2i-1\right.}$, *p* represents the past time vector, and *f* represents the future time vector. By extending the output equation in the system state Equation (5), a new extended output matrix can be obtained as

(8) $$Y_{f}=\Gamma_{f}X_{k}+H_{f}U_{f}+G_{f}E_{f}.$$

Γ_f_ is the observable matrix of the system and can be represented as

(9) $$\Gamma_{f}={\left[C\;\;CA\;\;\cdots \;\;CA^{f-1}\right]}^{T},$$

*H_f_* and *G_f_* are deterministic triangular Toeplitz matrices and stochastic triangular Toeplitz matrices, respectively, which can be represented as

(10) $$H_{f}=\left[\begin{array}{cccc} D & 0 & \cdots & 0\\ CB & D & \cdots & 0\\ \vdots & \vdots & \vdots & \vdots \\ CA^{f-2}B & CA^{f-3}B & \cdots & D \end{array}\right],$$

(11) $$G_{f}=\left[\begin{array}{cccc} I & 0 & \cdots & 0\\ CK & I & \cdots & 0\\ \vdots & \vdots & \vdots & \vdots \\ CA^{f-2}K & CA^{f-3}K & \cdots & I \end{array}\right],$$

It is defined that $X_{f}=L_{w}W_{p}+A_{K}^{r}X_{p}$, $A_{k}=A-KC$, $B_{k}=B-KC$; therefore, when *p* is sufficiently large, we can obtain $A_{K}^{p}\approx 0$. Thus, it can be inferred that

(12) $$Y_{f}=\Gamma_{f}L_{w}W_{p}+H_{f}U_{f}+G_{f}E_{f}.$$

where $W_{p}={\left[\begin{array}{cc} Y_{p}^{T} & U_{p}^{T} \end{array}\right]}^{T}$. By defining the matrix $O_{i}=\Gamma {\overset{\wedge }{X}}_{f}$, on which SVD decomposition is performed, we can obtain

(13) $$O_{i}=\Gamma_{f}L_{w}W_{p}=USV^{T}=U_{n}S_{n}V_{n}^{T},$$

Thus, the state sequence can be calculated as

(14) $$X_{i}=S_{n}^{1/2}V_{n}^{T},$$

By using the least squares method, the calculation results of the parameter matrix can be obtained as

(15) $$\left[\begin{array}{cc} \hat{A} & \hat{B}\\ \hat{C} & \hat{D} \end{array}\right]=\underset{A,B,C,D}{\min}{\left\|\left[\begin{array}{c} \hat{X}(k+1)\\ Y(k) \end{array}\right]-\left[\begin{array}{cc} A & B\\ C & D \end{array}\right]\left[\begin{array}{c} \hat{X}(k)\\ U(k) \end{array}\right]\right\|}_{F}^{2},$$

Thus, the state space matrix of the system can be identified.

Disturbances in signals, such as system uncertainty, noise interference, sensor accuracy limitations, data loss, and environmental factors, can lead to many difficulties in fault diagnosis, such as masking or distortion of feature information, increased bias in fault models, and reduced reliability of diagnostic results. Therefore, considering the impact of signal disturbance factors on fault diagnosis is of great significance. To suppress the influence of disturbances on the accuracy of fault diagnosis, a data-driven approach is adopted to introduce a prediction time domain of *m*. Based on the input data $\left\{x_{0},x_{1},\ldots ,x_{2m-1}\right\}$ of the system between time $\left\{0,1,\ldots ,2m-1\right\}$ and the output data $\left\{y_{0},y_{1},\ldots ,y_{m-1}\right\}$ at time $\left\{0,1,\ldots ,m-1\right\}$, the output at time $\left\{m,m+1,\ldots ,2m-1\right\}$ is predicted. To clarify the horizon definitions, the observation horizon and the prediction horizon are both set to length *m*. Specifically, at any given time step, the past horizon covers time indices 0 to *m* − 1, containing known system input/output data that provide historical information for establishing the initial state; the prediction horizon covers time indices *m* to 2*m* − 1, over which the model predicts the system output based on the past-horizon data and the inputs at these future instants. Consequently, the total data window used to construct the prediction model spans 2*m* consecutive samples from time 0 to 2m − 1. This symmetric setting enables the algorithm to fuse *m* historical data points to predict the next *m* steps in the rolling optimization, thereby achieving dynamic filtering and disturbance suppression in the time domain. The state value of the motor model fused with past and predicted time-domain data can be represented as

(16) $$\hat{y}(m)=L_{x}\cdot x_{f}(m)+L_{w}\cdot w_{p}(m),$$

where $\hat{y}(m)={\left[{\hat{y}}_{m}\;{\hat{y}}_{m+1}\;\ldots \;{\hat{y}}_{2m-1}\right]}^{T}$ is the output value of the model state, $w_{p}(m)$ is the input data in the past time domain, $x_{f}(m)$ is the input data in the prediction time domain, and $L_{x}$ and $L_{w}$ are the identified parameter matrices. These are further defined as $w_{p}(m)=\left[\begin{array}{c} x_{p}(m)\\ y_{p}(m) \end{array}\right]$, $x_{p}(m)=\left[\begin{array}{c} x_{m-i}\\ x_{m-i+1}\\ \vdots \\ x_{m-1} \end{array}\right]$, $y_{p}(m)=\left[\begin{array}{c} y_{m-i}\\ y_{m-i+1}\\ \vdots \\ y_{m-1} \end{array}\right]$, $x_{f}(m)=\left[\begin{array}{c} x_{m}\\ x_{m+1}\\ \vdots \\ x_{m+i-1} \end{array}\right]$, where subscript *f* represents the predicted time domain and subscript *p* represents the past time domain. In an ideal situation, the predicted value of the model state should be exactly the same as the measured value of the model. Therefore, fault diagnosis can be achieved by comparing the deviation between the predicted value of the model state and the measured value.

The value of *m* should match the effective time constant and sampling period of the system. *m* that is too small leads to insufficient predictive information and poor disturbance suppression, while *m* that is too large introduces excessive historical data, reducing the responsiveness to fast-changing fault signals. In practice, the optimal *m* was determined via cross-validation and model-order selection criteria.

The prediction horizon *m* determines the length of historical data used for state prediction, and its optimal value is theoretically related to the dominant time constant *τ* and sampling period *T_s_* of the motor system. For a typical permanent magnet brushless DC motor, the dominant time constant is determined by the electrical time constant *τ_e_* = *L/R* and mechanical time constant *τ_m_* = *J_m_/b*. In most direct-drive in-wheel motor applications, *τ_e_* ≤ *τ_m_*, so *τ_m_* dominates the system dynamic response. To ensure the predicted output covers the main dynamic process of the motor, *m* should satisfy *m·T_s_* ≈ (0.8–1.2)*τ_m_*, where 0.8–1.2 is the coefficient covering the 10–90% rise time of the step response. For the motor studied, *τ_m_* = *J_m_/b* = 0.2 s and *T_s_* = 0.001 s. The theoretical range of *m* is 160–240 in discrete steps, but excessive *m* introduces more noise samples, so this theoretical range needs to be adjusted considering the noise suppression requirement of subspace identification. Therefore, a tuning method based on cross-validation is recommended for practical applications.

This fusion strategy, referred to as the hybrid data–mechanistic approach throughout this paper, leverages the mechanistic model to provide the physical structure and main dynamic characteristics, while the data-driven subspace identification optimizes and supplements it using actual operational data, thereby reducing identification difficulty and improving efficiency.

### 3.2. Fault Residual Generation and Anti-Interference Filtering Under Signal Disturbances

When a sensor failure occurs, the output value of the motor model system state can be expressed in the following form

(17) $$y(m)=[L_{x}\;L_{w}]\left[\begin{array}{c} x_{f}(m)\\ w_{p}(m) \end{array}\right]+{\tilde{G}}_{f}\cdot f(m),$$

where $y(m)={\left[y_{m}\;y_{m+1}\;\ldots \;y_{2m-1}\right]}^{T}$, ${\tilde{G}}_{f}$ is the fault transition matrix, and f(m) represents sensor failure. Considering that the current and voltage sensor data of the motor system are affected by external disturbances, the actual sensor information output by the system can be represented in the following form

(18) $$y(m)=[L_{x}+\Delta L_{x}\;L_{w}+\Delta L_{w}]\left[\begin{array}{c} x_{f}(m)\\ w_{p}(m) \end{array}\right]+{\tilde{G}}_{f}\cdot f(m).$$

Combining Equations (16) and (18), the state prediction error $e(m)={\left[e_{m}\;e_{m+1}\;\ldots \;e_{m+i-1}\right]}^{T}$ that accounts for system disturbances can be expressed as

(19) $$e(m)=y(m)-\hat{y}(m)=y(m)-[L_{x}\;L_{w}]\left[\begin{array}{c} x_{f}(m)\\ w_{p}(m) \end{array}\right]=[\Delta L_{x}\;\Delta L_{w}]\left[\begin{array}{c} x_{f}(m)\\ w_{p}(m) \end{array}\right]+{\tilde{G}}_{f}\cdot f(m),$$

It is known that error e(m) includes both system disturbance outputs and sensor faults. Therefore, in the process of fault diagnosis, in order to improve the accuracy of fault diagnosis, it is necessary to extract error information that does not contain system disturbances. Further, to improve the reliability of fault residual signals, an adaptive filter is designed to generate residual signals r(m) and can be denoted as

(20) $$r(m)=F\cdot [y(m)-\hat{y}(m)]=F\cdot (\Delta L\left[\begin{array}{c} x_{f}(m)\\ w_{p}(m) \end{array}\right]+{\tilde{G}}_{f}\cdot f(m)),$$

where *F* is the filtering gain and can be expressed as $\Delta L=[\Delta L_{x}\;\Delta L_{w}]$, and both *x_f_*(*m*) and *w_p_*(*m*) are column vectors.

The signal disturbance can affect the representational capability of residual signals, thereby reducing the sensitivity of fault diagnosis and even potentially increasing the false alarm rate. To suppress the negative impact of signal disturbance, a residual signal anti-interference filter is constructed to improve the reliability of the residual signal in the face of influence. According to Equation (20), when the system is in normal working condition, the error *e*(*m*) is caused by system disturbance and can be written as $e(m)=\Delta L{\left[\begin{array}{cc} X_{f}(m) & W_{p}(m) \end{array}\right]}^{T}$. Because the disturbance effects of the system cannot be directly simulated using a known function, the analysis of *ΔL* is based on the data at time *m* when the system is working normally, together with the data before and after time *i* and the prediction error data E*. Then, *ΔL* can be obtained by solving the following minimization index function

(21) $$\underset{\Delta L_{x},\Delta L_{w}}{\min}{\left\|E^{*}-[\Delta L_{x}\;\Delta L_{w}]\left[\begin{array}{c} X_{f}^{*}\\ W_{p}^{*} \end{array}\right]\right\|}_{F}^{2},$$

where $E^{*}=\left[\begin{array}{cccc} e_{m-i-j^{*}+2}^{*} & e_{m-i-j^{*}+3}^{*} & \ldots & e_{m-i+1}^{*}\\ e_{m-i-j^{*}+3}^{*} & e_{m-i-j^{*}+4}^{*} & \ldots & e_{m-i+2}^{*}\\ \vdots & \vdots & \ddots & \vdots \\ e_{m-j^{*}+1}^{*} & e_{m-j^{*}+2}^{*} & \ldots & e_{m}^{*} \end{array}\right]$, $X_{p}^{*}=\left[\begin{array}{cccc} x_{m-2i-j^{*}+2} & x_{m-2i-j^{*}+3}^{*} & \ldots & x_{m-2i+1}^{*}\\ x_{m-2i-j^{*}+3}^{*} & x_{m-2i-j^{*}+4}^{*} & \ldots & x_{m-2i+2}^{*}\\ \vdots & \vdots & \ldots & \vdots \\ x_{m-i-j^{*}+1}^{*} & x_{m-i-j^{*}+2}^{*} & \ldots & x_{m-i}^{*} \end{array}\right]$, $E_{p}^{*}=\left[\begin{array}{cccc} e_{m-2i-j^{*}+2}^{*} & e_{m-2i-j^{*}+3}^{*} & \ldots & e_{m-2i+1}^{*}\\ e_{m-2i-j^{*}+3}^{*} & e_{m-2i-j^{*}+4}^{*} & \ldots & e_{m-2i+2}^{*}\\ \vdots & \vdots & \ddots & \vdots \\ e_{m-i-j^{*}+1}^{*} & e_{m-i-j^{*}+2}^{*} & \ldots & e_{m-i}^{*} \end{array}\right]$, $X_{f}^{*}=\left[\begin{array}{cccc} x_{m-i-j^{*}+2} & x_{m-i-j^{*}+3}^{*} & \ldots & x_{m-i+1}^{*}\\ x_{m-i-j^{*}+3}^{*} & x_{m-i-j^{*}+4}^{*} & \ldots & x_{m-i+2}^{*}\\ \vdots & \vdots & \ldots & \vdots \\ x_{m-j^{*}+1}^{*} & x_{m-j^{*}+2}^{*} & \ldots & x_{m}^{*} \end{array}\right]$, $W_{p}^{*}=\left[\begin{array}{c} X_{p}^{*T}\\ E_{p}^{*T} \end{array}\right]$, $j^{*}$ is the matrix length. In addition, *ΔL* can be expressed as

(22) $$\Delta L=\left[\begin{array}{cc} \Delta L_{x} & \Delta L_{w} \end{array}\right]=\left[\begin{array}{cc} R_{\Delta 31}^{*} & R_{\Delta 32}^{*} \end{array}\right]V_{q\Delta }^{*}\left[\begin{array}{cccc} {\sigma_{1\Delta }^{*}}^{-1} & 0 & \ldots & 0\\ 0 & {\sigma_{2\Delta }^{*}}^{-1} & \ldots & 0\\ \vdots & \vdots & \ddots & \vdots \\ 0 & 0 & \ldots & {\sigma_{q\Delta }^{*}}^{-1} \end{array}\right]{U_{q\Delta }^{*}}^{T},$$

**Assumption 1.**  *The disturbance sensitivity matrix ΔL = [ΔL_x_ ΔL_w_] is row full-rank, which guarantees that all singular values in its SVD are positive and invertible. This assumption holds for the hub motor system under normal operation, as the disturbance coupling between current, speed, input voltage. and load torque is linearly independent.*

**Assumption 2.**  *The fault transfer matrix* ${\tilde{G}}_{f}$ *is column full-rank, ensuring that fault features are distinguishable from disturbance components. This is satisfied for the gain-type sensor faults validated in the experimental section, where the fault acts directly on the output channel without coupling to disturbance terms.*

For clarity, the dimensions of all matrices involved in the filter derivation are explicitly defined:

(1) Prediction error vector $e\left(m\right)\in R^{n_{y}\cdot m\times 1}$, where *n_y_* = 2 denotes the number of output variables for current and motor speed, and *m* is the prediction horizon length.
(2) Disturbance sensitivity matrix $\Delta L=\left[\begin{array}{cc} \Delta L_{x} & \Delta L_{w} \end{array}\right]\in R^{n_{y}\cdot m\times \left(n_{x}\cdot m+(n_{y}+n_{u})\cdot i\right)}$, where *n_x_* = 2 is the number of system states, *n_u_* = 2 is the number of input variables for terminal voltage and load torque, and *i* is the number of past time blocks in the subspace identification.
(3) Filter gain matrix $F\in R^{n_{y}\cdot m\times n_{y}\cdot m}$, which is dimension-compatible with *e*(*m*) to ensure the residual vector maintains the same dimension as the prediction error.
(4) Fault transfer matrix ${\tilde{G}}_{f}\in R^{n_{y}\cdot m\times n_{f}}$, where *n_f_ =* 1 for single-sensor gain faults.

The purpose of anti-interference filtering design is to minimize the impact of system disturbances on residual signals and amplify fault signals as much as possible. Therefore, the residual signal needs to satisfy the condition $J_{m}=\max\frac{{\left\|F\cdot {\tilde{G}}_{f}\right\|}_{2}}{{\left\|F\cdot \Delta L\right\|}_{2}}$, where ${\left\|\cdot \right\|}_{2}$ denotes the induced 2-norm of a matrix, which satisfies the submultiplicativity property ${\left\|AB\right\|}_{2}\leq {\left\|A\right\|}_{2}{\left\|B\right\|}_{2}$ for any dimension-compatible matrices *A* and *B*, and is naturally compatible with SVD decomposition.

When *J_m_* reaches its optimal value, the filter is considered to have met the requirements. By performing singular value decomposition on Equation (22), we can obtain

(23) $$\Delta L=[\Delta L_{x}\;\;\Delta L_{w}]=[\Delta R_{31}\;\;\Delta R_{32}]V_{\Delta f}\left[\begin{array}{cccc} {\sigma_{\Delta f_{1}}}^{-1} & 0 & \cdots & 0\\ 0 & {\sigma_{\Delta f_{2}}}^{-1} & \cdots & 0\\ \vdots & \vdots & \ddots & \vdots \\ 0 & 0 & \cdots & {\sigma_{\Delta f_{m}}}^{-1} \end{array}\right]U_{\Delta f}^{T},$$

**Proof.**  Because $\Delta L=[\Delta L_{x}\;\;\Delta L_{w}]$ can be decomposed by the SVD method, *ΔL* can be rewritten as follows

(24) $$\Delta L=[\Delta L_{x}\;\;\Delta L_{w}]=U_{\Delta }\Sigma_{\Delta }V_{\Delta }^{T},$$

where $\Sigma_{\Delta }=[diag(\sigma_{\Delta 1},\sigma_{\Delta 2},\ldots ,\sigma_{\Delta n})]$, and both $U_{\Delta }$ and $V_{\Delta }$ are orthogonal matrices.

To decouple the fault amplification term from the disturbance suppression term in the optimization objective, we parameterize the filter gain as $F=F_{1}S_{\Delta }^{-1}U_{\Delta }^{T}$, where $F_{1}\in R^{n_{y}\cdot m\times n_{y}\cdot m}$ is an arbitrary non-zero matrix. This parameterization is designed to normalize the disturbance term in the residual. If $S_{\Delta }=diag(\sigma_{\Delta 1},\sigma_{\Delta 2},\ldots ,\sigma_{\Delta n})$ for the condition $\forall F_{1}\neq 0$, the following relationship of the denominator of *J_m_* can be satisfied

(25) $${\left\|F\cdot \Delta L\right\|}_{2}={\left\|F_{1}S_{\Delta }^{-1}U_{\Delta }^{T}\cdot U_{\Delta }\Sigma_{\Delta }V_{\Delta }^{T}\right\|}_{2}={\left\|F_{1}S_{\Delta }^{-1}\Sigma_{\Delta }V_{\Delta }^{T}\right\|}_{2}={\left\|F_{1}I_{n_{y}\cdot m}V_{\Delta }^{T}\right\|}_{2}={\left\|F_{1}\right\|}_{2}.$$

Equation (24) holds for two reasons: first, $S_{\Delta }^{-1}\Sigma_{\Delta }=I_{n_{y}\cdot m}$ because all non-zero singular values of *ΔL* are retained in *S_Δ_* under Assumption 1; second, multiplying by an orthogonal matrix *V_Δ_* does not change the spectral norm of a vector.

Substituting the parameterized *F* into the numerator of *J_m_* and applying the submultiplicativity property of the induced 2-norm gives

(26) $${\left\|F\cdot {\tilde{G}}_{f}\right\|}_{2}={\left\|F_{1}S_{\Delta }^{-1}U_{\Delta }^{T}\cdot {\tilde{G}}_{f}\right\|}_{2}\leq {\left\|F_{1}\right\|}_{2}\cdot {\left\|S_{\Delta }^{-1}U_{\Delta }^{T}\cdot {\tilde{G}}_{f}\right\|}_{2}.$$

Combining the results of the numerator and denominator, the upper bound of *J* is ${\left\|S_{\Delta }^{-1}U_{\Delta }^{T}\cdot {\tilde{G}}_{f}\right\|}_{2}$, which is achieved if and only if $F_{1}=I_{n_{y}\cdot m}$ (the $n_{y}\cdot m$-order identity matrix). This is because setting *F_1_* = *I* aligns the filter with the right singular vector of $S_{\Delta }^{-1}U_{\Delta }^{T}\cdot {\tilde{G}}_{f}$, eliminating unnecessary norm attenuation caused by arbitrary choices of *F_1_*. □

Therefore, the optimal filter gain is $F=S_{\Delta }^{-1}U_{\Delta }^{T}$. By substituting the optimal *F* into Equation (20), the filtered residual signal can be obtained as

(27) $$r(m)=F\cdot e(m)=S_{\Delta }^{-1}U_{\Delta }^{T}\cdot [y(m)-\hat{y}(m)],$$

By predicting the rolling adjustment in the time domain, dynamic and reliable estimation of residual signals under disturbance conditions can be achieved.

### 3.3. Adaptive Threshold Design Considering System Interference

Based on the identified model and residual filtering results, the sensor fault diagnosis scheme of vehicle’s direct-drive motor system can be represented as shown in Figure 2. In normal operation, the system is fault-free, but various signal disturbances such as noise, model uncertainties, and environmental factors are inevitably present. The proposed method employs an adaptive residual filter that is specifically designed to suppress the influence of these disturbances on the residual signal. Consequently, the resulting residual maintains a low magnitude under normal conditions. However, due to the inherent limitations of disturbance modeling and the presence of unmodeled dynamics, the residual does not strictly approach zero. When a sensor fault occurs, the fault-induced deviation is amplified by the filter’s design objective of maximizing fault sensitivity, leading to a significant increase in the residual magnitude. This deviation from the normal low level is then used to trigger a fault alarm when it exceeds the adaptive threshold. Based on this, a threshold is usually set to evaluate the residual signal.

In order to reduce the inaccuracy of the model itself and the adverse effects caused by environmental factors that are difficult to model, the maximum likelihood ratio method can be used to evaluate the residual signal, thereby avoiding the problems of abnormal residual signals and false alarms caused by system interference and model uncertainty. Under the null hypothesis *H_0_* (sensor fault-free), the filtered residual *r*(*m*) is assumed to be a zero-mean Gaussian random variable. Its variance *σ^2^*(*m*) is estimated from a representative set of fault-free operational data and reflects the current disturbance intensity. Among them, the expression of the fault determination factor based on maximum likelihood ratio estimation [52,53] is as follows

(28) $$J=\frac{r{\left(m\right)}^{2}}{\sigma {\left(m\right)}^{2}},$$

where *r*(*m*) is the residual value, $\sigma {\left(m\right)}^{2}$ is the residual when there is no sensor fault, and *J* satisfies $J\sim \chi^{2}(1)$. This chi-square property is a direct consequence of the normalization: since *r*(*m*)*/σ*(*m*)-*N*(0,1) under *H_0_*, its square follows *χ^2^*(1). The threshold *T_r_* is then the upper-tail critical value of *χ^2^*(1) corresponding to the prescribed false alarm rate *P_f_*. If the required false alarm rate *P_f_* is known, the value of the threshold *T_r_* can be obtained by the following equation

(29) $$P\left\{J>T_{r}\left|s=0\right.\right\}=P_{f},$$

where *s* = 0 indicates not exceeding the threshold, and *s* = 1 indicates exceeding the threshold. The adaptive threshold is defined as the critical value of the chi-square distribution at a preset false alarm rate.

Based on the known false alarm rate, the threshold can be determined by searching the chi-square distribution table. The logic of fault detection is

(30) $$s=\left\{\begin{array}{c} 0,J<T_{r}\\ 1,J>T_{r} \end{array}\right..$$

The term “adaptive” reflects that the threshold is not a fixed constant but is derived from the estimated variance *σ^2^*(*m*), which captures the time-varying disturbance level of the system. As operating conditions change, *σ^2^*(*m*) can be updated from recent fault-free data, allowing the threshold to automatically adjust while maintaining the preset *P_f_*.

It is worth noting how the present approach relates to the hybrid strategies [39,40,41]. While these strategies also integrate physical models and data-driven techniques, they typically either use a physics-based model primarily to augment the input/feature space for a downstream classifier, or train a neural network to directly serve as both the system model and the fault decision-maker. In contrast, the method proposed here adopts a two-stage architecture that is clearly separated: data-driven subspace identification is first used to obtain an explicit, interpretable discrete state-space model, upon which an adaptive residual filter is then independently designed to suppress disturbance-induced residual distortion, followed by an adaptive threshold that is based on the likelihood ratio. This coordinated-yet-decoupled arrangement allows the residual dynamics to be analyzed analytically and the threshold to be calibrated statistically. Furthermore, unlike the approach in [40], which constructs noisy/disturbance-injected training data, the proposed method directly exploits actual measured fault-free experimental data, avoiding the distribution shift between training and deployment.

## 4. Experimental Verification by Motor Bench Test and Real Vehicle Test

### 4.1. Verification of Model Identification Results

The structure of the experimental bench is shown in Figure 3. The direct-drive wheel-hub motor, speed/torque sensor, and magnetic powder brake are securely installed in their respective positions on the bench using specific clamping devices. Connect them to each other through a coupling and adjust them to the same vertical height to meet the requirements of coaxiality. Specifically, the direct-drive in-wheel motor is a XDY2011-10/72 prototype, with a rated voltage of 72 V, rated power of 5 kW, rated torque of 20 N·m, peak torque of 70.94 N·m, maximum speed of 4227 rpm, and air-cooled heat dissipation. The speed/torque sensor is a rotary torque transducer mounted on the coupling shaft, and the magnetic powder brake is used as the load device. Coaxiality among the three components is guaranteed by precision couplings and height-adjustable supports.

The operation of the direct-drive wheel-hub motor is precisely controlled by the motor controller. The control signal emitted by the motor controller is within the range of 1 V to 3 V, and this signal range corresponds to the torque response range generated by the motor. To monitor the working condition of the direct-drive motor in real time, voltage sensors and current sensors were used during the experiment. These two sensors can accurately record the relevant data of the input voltage and input current of the motor controller. The voltage and current sensors are Hall-effect isolated sensors, and the sampling frequency of the data acquisition system is set to 1 kHz to ensure that both steady-state and transient characteristics are accurately captured.

During the experiment, the Decaron battery testing system acts as a constant voltage source, providing the motor with a continuous and stable supply of the required voltage. At the same time, the magnetic powder brake is responsible for applying corresponding loads to simulate actual working conditions. The speed/torque sensor works continuously to measure and record the specific values of the speed and torque presented by the wheel-hub motor under different given control signal conditions. During this process, the initial voltage of the motor-controller control signal is set to 1 V, and then gradually increases by 0.2 V each time until the voltage of the control signal reaches 3 V.

The data acquisition system operates at a sampling frequency of 1000 Hz, which covers the dynamic response bandwidth of the motor. In the subspace identification procedure, the system order is set to 4, determined by performing singular value decomposition on the Hankel matrix and retaining principal components accounting for at least 90% of the total energy. The number of block rows *i* in the Hankel matrix is 20, satisfying *i* > 2*n* and *i* ≤ *N*/3, where *N* = 1000 is the number of data samples per experiment. To balance computational efficiency and statistical sufficiency, the number of block columns is set to *j* = 500, which satisfies the theoretical constraint *j* ≤ *N* − 2*i* + 1 = 961.

To simulate the electronic thermal noise and electromagnetic interference commonly present in actual sensor measurements, additive white Gaussian noise was injected into the current and voltage sensor signals. The noise intensity was controlled by the signal-to-noise ratio (SNR), which was set to 20 dB, the default value used in this study. This value was chosen based on typical noise levels in electric vehicle drive-motor sensor environments, and was validated to ensure that the fault features remain distinguishable while sufficiently challenging the anti-interference capability of the proposed algorithm.

Accurate and reliable model state identification results are the foundation for accurate fault diagnosis. Therefore, it is necessary to first verify the results of the motor-model state identification. To verify the practical effectiveness of the proposed subspace identification algorithm for motor models considering signal disturbances, the identification of motor-model states under the influence of noise disturbances and model uncertainty disturbances was carried out successively. The state identification results of the motor model under the influence of noise are shown in Figure 4. The identification results of the motor-model state include the motor-current and motor-speed state values. Overall, the identified model can effectively track the actual state curves of current and speed. The consistency between the model state values and the actual measured values is relatively high in terms of the trend of the model state curve and the actual amplitude size. However, after injecting noise interference into the model, the resulting model state variables showed significant fluctuations. As shown in Figure 4a, under noise interference, the local magnification reveals that although the overall trend of the motor current curve remains largely unchanged, its amplitude fluctuates substantially, reducing the accuracy of model state identification. Additionally, as shown in Figure 4b, the motor speed curve also showed the same situation under the influence of noise interference. The motor speed also exhibited substantially greater amplitude fluctuations under noise interference, and the speed curve even showed noticeable distortion. These results demonstrate that the proposed subspace identification algorithm provides relatively accurate model identification results. However, under the influence of additional noise interference, the state values of the identified model may have a certain degree of deviation. This deviation, caused by noise interference, can affect the characterization of residuals, which can limit the effectiveness of sensor fault diagnosis. The above analysis further demonstrates the necessity of the proposed fault residual generation and residual signal anti-interference filtering method for mitigating signal disturbances.

The identification results of residual filtering under the influence of noise interference are shown in Figure 5. As shown in the figure, when the residual signal is not affected by noise interference, the overall curve of the residual signal trends toward 0 and the fluctuation range is relatively small. When the residual signal is disturbed by noise and no filtering is applied, the overall residual signal exhibits significant fluctuations, and many sampling points show severe amplitude deviations. In this case, if the proposed filtering method is used, the overall amplitude fluctuation of the residual signal obtained is well suppressed, and it converges again and stabilizes at zero. The overall residual variation curve is in good agreement with the parameter curve without noise interference. This performance demonstrates that the proposed residual signal anti-interference filtering algorithm can effectively suppress the abnormal influence of noise interference on the residual signal, thereby providing an improved representation of the actual residual signal.

To further evaluate the robustness of the proposed method under varying noise intensities, comparative experiments were conducted at SNR levels of 15 dB, 20 dB, 25 dB, and under noise-free conditions. Table 1 presents the root mean square error (RMSE) of current and speed state identification under these conditions. As expected, the identification error increases with decreasing SNR. When the SNR drops from 25 dB to 15 dB, the current RMSE rises from 0.15 A to 0.38 A, and the speed RMSE increases from 16.8 rpm to 41.5 rpm. Even under relatively strong noise with the SNR at 15 dB, the proposed method maintains acceptable identification accuracy, with RMSE increments remaining within a controllable range, demonstrating satisfactory noise immunity. At the default SNR of 20 dB, the identification errors are substantially smaller than the fluctuation amplitudes induced by noise, providing a reliable foundation for subsequent residual filtering and fault diagnosis.

### 4.2. Sensitivity Analysis of Prediction Horizon

The prediction horizon *m* balances the utilization of historical dynamic information and the suppression of measurement noise in subspace identification. As discussed in Section 3.1, the theoretical range of *m* for the studied motor is 160~240 based on the mechanical time constant, but this range is derived for full dynamic process coverage. In the proposed method, *m* mainly affects the disturbance suppression performance of the residual filter rather than the full state tracking, so a smaller *m* is preferred to avoid introducing redundant noise. To determine the optimal *m* for the proposed fault diagnosis framework, a five-fold cross-validation procedure is adopted:

1. Divide the collected normal operation dataset into five equal subsets.
2. For each candidate *m* ∈ {5, 8, 10, 12, 15, 20}, train the subspace identification model and residual filter on four subsets, and calculate the root mean square error (RMSE) of state identification and the standard deviation of filtered residuals on the remaining subset.
3. Repeat step 2 for all five subsets, and take the average RMSE and residual standard deviation as the evaluation metrics.
4. Select the *m* that minimizes both RMSE and residual standard deviation as the optimal value.

The choice of the prediction horizon *m* directly affects the state prediction accuracy and residual filtering performance of the subspace identification model. To quantitatively evaluate its impact, six values of *m* (5, 8, 10, 12, 15, 20) were tested under identical noise disturbance conditions with the SNR at 20 dB. The RMSE of the current and speed states was used as the metric for identification accuracy, while the mean *μ_r_* and standard deviation *σ_r_* of the filtered residuals served as indicators of residual quality.

Figure 6 shows the RMSE variation curves for current and speed under different *m* values. The horizontal axis denotes the prediction horizon *m*, and the vertical axis represents RMSE. When *m* = 5, the current RMSE is approximately 0.48 A and the speed RMSE is about 52 rpm, both relatively high. As *m* increases to the range of 8–12, both RMSE values decrease rapidly and stabilize, reaching minima at *m* = 10. When *m* > 15, the RMSE increases slightly, indicating that an excessively large prediction horizon introduces redundant historical information and impairs the model’s ability to track rapidly changing signals.

Table 2 summarizes the statistical properties of the filtered residuals for different *m* values. At *m* = 10, the residual mean and standard deviation are 0.002 and 0.015, respectively, both of which are the smallest, demonstrating the best disturbance suppression performance. Based on the trade-off between identification accuracy and residual quality, *m* = 10 was selected as the default prediction horizon in this study, and it exhibited robust performance across all bench and real-vehicle experiments.

The above analysis confirms that prediction horizons in the range of 8–12 provide satisfactory robustness. The selection of the prediction horizon is crucial. If *m* is too small, the residual model cannot fully capture the system dynamics, leading to a larger RMSE and reduced fault detection sensitivity. If *m* is too large, the RMSE also increases, which may cause more false positives in fault diagnosis. Therefore, the prediction horizon 10 was chosen as a compromise that balances prediction accuracy and dynamic response speed.

It should be emphasized that the optimal *m* may vary for different motor systems due to differences in power ratings, load characteristics, and sampling frequencies. For systems with faster dynamic response (smaller *τ*), a smaller *m* is recommended to avoid excessive historical data smoothing that masks fast-changing fault features. For systems with lower sampling frequencies, *m* should be increased appropriately to ensure sufficient data points for subspace identification. The cross-validation procedure described above provides a standardized and reproducible way to determine *m* for various applications, which only requires normal operation data without fault labels.

### 4.3. Verification of Fault Detection Results

To comprehensively reflect the effectiveness of the proposed data-driven model identification method and residual signal anti-interference filtering algorithm under various types of interference, further validation was conducted on an actual vehicle testing platform under uncertain interference conditions. Figure 7 shows the architecture of the vehicle-mounted direct-drive motor testing platform based on a chassis dynamometer system.

The vehicle in this experiment incorporates distributed-drive electric chassis architecture, with wheel-hub motors installed at each wheel as direct-drive motors. By conducting vehicle chassis dynamometer experiments, relevant driving data of permanent magnet DC brushless motors in actual vehicle application scenarios can be collected. This experimental platform is built using the D2P MotoHawk rapid development prototype system. The D2P ECU hardware operates at 12 V DC and provides 30 analog inputs, 4 low-frequency inputs, 4 VR frequency inputs, 10 low-side PWM outputs, and 2 CAN channels, offering sufficient interfaces for sensor data acquisition and control signal generation. A PWM-to-analog conversion module (supply voltage 9–24 V, output 0–5 V/0–10 V) is used to achieve input/output matching between the motor controller and the rapid prototyping hardware. This integrated development environment relies on the hardware architecture of mass-produced ECU and MATLAB/Simulink joint simulation technology, which can directly call industrial grade motor controller hardware resources to achieve rapid development of the drive system, eliminating the need for secondary development of underlying hardware. During the testing process, a motor control algorithm model based on the MotoHawk development environment was used to send specific voltage excitation signals to the actuator.

To comprehensively evaluate the proposed method across the motor’s full operational envelope, the experiments were designed to simulate dynamic responses under various constant power demand conditions, covering the transition from start-up to steady-state, constant-speed operation. This approach ensures that the fault characteristics are captured under representative real-world scenarios rather than being limited to a single standard driving cycle. The composite sensing network configured for the drive system includes current/voltage sensing modules located on the powertrain, which can obtain real-time parameters of the controller power bus. The current- and voltage-sensing modules adopt Hall-effect isolated transducers with measurement accuracy better than ±1%, while the speed detection unit is based on gear pulse counting and the steering-angle/torque sensors provide analog outputs conditioned through the D2P I/O module. A speed detection unit based on the principle of gear pulse counting is used to measure the angular velocity of the motor. The torque analysis system equipped inside the chassis dynamometer uses mechanical parameter conversion to obtain the numerical value of mechanical torque. All dynamic parameters are synchronously collected through the CAN bus protocol via multiple channels. At the same time, the onboard VehicleSPY network analyzer is used to perform dual recording and cross validation of controller command signals and sensor data. All dynamic parameters are time-aligned and synchronously acquired through the two CAN channels of the D2P ECU at a bus baud rate of 500 kbps, ensuring consistent timestamps across current, voltage, speed, and torque signals during fault diagnosis.

The identification results of the model state under the influence of uncertainty interference are shown in Figure 8. As shown in Figure 8, the overall trends of the motor current and speed curves obtained from the identification model closely follow the actual corresponding measured values. However, based on the local magnified image, it can be seen that under the influence of uncertainty interference, the model state variables are significantly affected, and both the current curve and the speed curve show deviations and oscillations in the overall trend. This also indicates that under the influence of uncertainty interference, further filtering methods are needed to obtain residual results based on model identification of states.

The identification results of residual filtering under the influence of uncertainty interference are shown in Figure 9. As shown in the figure, in the absence of interference, the overall residual curve still exhibits a fluctuating pattern around zero. When there is uncertainty interference and no filtering is used, there is a significant high-frequency oscillation and amplitude deviation problem in the residual value. Especially during the initial start-up stage of the motor, the motor current responds rapidly and increases sharply, while the amplitude of the motor state residual also changes substantially. At the same time, due to the relatively balanced increase and change in motor speed relative to current, the residual amplitude in the motor speed residual curve also shows an increasing trend with an increase in speed amplitude after the motor starts, ultimately deviating significantly from the residual size in the absence of interference. In contrast, when there is uncertainty interference and the anti-interference filtering algorithm is used, the oscillation and deviation problems in the residual filtering result in well-suppressed curves of current and speed. The filtered residual curve is in good agreement with the residual obtained without interference, indicating that the proposed filtering method effectively mitigates the adverse effects of interference and improves the residual’s representation of fault information and sensitivity to faults.

On the basis of verifying the model identification and residual filtering effects, the effectiveness of sensor fault diagnosis is further confirmed. In the experimental verification, current and speed sensor failure were set to occur at the 40th second. At this time, a multiple gain fault was used to simulate the actual sensor failure, where the fault function is $f(t)=\left\{\begin{array}{c} 0,\;t\leq 40s\\ 5y\left(m\right),\;t>40s \end{array}\right.$. To statistically validate the reliability of the proposed method, the fault injection experiment was repeated 10 times under identical operating conditions.

Figure 10 shows the fault diagnosis results when the current sensor malfunctions. When the current sensor malfunctions, causing the measured current to become abnormal and increase sharply, the residual value calculated from the measured current and the filtered identification result also increases sharply. In this case, the maximum likelihood ratio of the residual current state will also increase substantially more than the estimated result. As shown in the figure, when the current sensor malfunctions, the maximum likelihood ratio estimation of the current state residual increases sharply and significantly exceeds the set adaptive threshold. In this case, the maximum likelihood ratio estimation of the residual speed state does not show significant changes due to the fault. Therefore, it can be determined that the current sensor has a fault, while the speed sensor has no fault. The introduction of maximum likelihood ratio effectively increases the sensitivity of residual information to fault factors after the occurrence of faults, thereby enhancing the effectiveness of fault diagnosis and avoiding misjudgments caused by unreasonable threshold selection.

Figure 11 shows the fault diagnosis when the speed sensor malfunctions. Similarly, as shown in the figure, for a fault in the speed sensor, the maximum likelihood ratio estimation of the residual current state does not show significant fluctuations due to the occurrence of the fault. Under the same conditions, the maximum likelihood ratio estimate of the residual speed state increases sharply when the fault occurs and significantly exceeds the preset threshold, clearly indicating a speed sensor malfunction.

### 4.4. Quantitative Comparison with Baseline Methods

To further validate the individual contributions of each technical module in the proposed method, four progressive methods were designed for quantitative comparison. All methods were evaluated under identical experimental conditions.

Method 1 employs standard N4SID subspace identification [14] without explicitly modeling noise and model uncertainty. No anti-interference filtering is applied to the residuals, and a fixed threshold determined by the 3*σ* rule based on fault-free residuals is used for fault decision-making. This represents the most basic implementation of subspace identification-based fault diagnosis. Method 2 incorporates the predictive time-domain rolling mechanism [21] into the N4SID identification process to account for signal disturbances, which is the same as in the proposed method. However, no anti-interference filtering is applied to the residuals, and a fixed threshold is still used. This configuration serves to validate the contribution of the disturbance-aware model identification module. Method 3 builds upon the second method by adding SVD-optimized residual anti-interference filtering [16], while retaining the fixed threshold for fault decision-making, thereby validating the contribution of the residual filtering module. Method 4 applies the sliding-mode observer [48] for state acquisition with generic residual anti-interference filtering. This represents a conventional model-driven fault diagnosis path that uses SMO for state estimation, with standard residual processing but lacking adaptive decision capability. Method 5 is CNN-LSTM [39] for state acquisition with a proposed maximum likelihood ratio-based adaptive threshold. This represents a conventional data-driven fault diagnosis path that uses CNN-LSTM for state estimation, with adaptive decision capability but lacking dedicated residual denoising. Method 6 is the proposed method, which further replaces the fixed threshold with a generalized likelihood ratio-based adaptive threshold, validating the contribution of the adaptive threshold module.

Three metrics are adopted for evaluation. The fault detection delay is defined as the time difference between fault injection and successful fault detection, measured in seconds. The false alarm rate is the percentage of sampling points for which the residual exceeds the threshold during the fault-free period. The missed detection rate is the percentage of sampling points in which the residual remains below the threshold during the fault period.

The comparative results of the six methods are presented in Table 3. The contribution of each technical module can be clearly observed. Comparing the first and second methods, introducing the predictive time-domain rolling mechanism to account for disturbances, reduces the detection delay from 0.48 s to 0.35 s (a 27% reduction), the false alarm rate from 12.3% to 8.2% (a 33% reduction), and the missed detection rate from 10.5% to 6.5% (a 38% reduction). This demonstrates that considering disturbances during the model identification stage significantly improves identification accuracy and consequently enhances downstream fault diagnosis performance. Comparing the second and third methods, adding residual anti-interference filtering, further reduces the detection delay from 0.35 s to 0.18 s (a 49% reduction), the false alarm rate from 8.2% to 3.6% (a 56% reduction), and the missed detection rate from 6.5% to 2.4% (a 63% reduction). This indicates that residual anti-interference filtering is a critical step for suppressing the impact of disturbances on residual signals, substantially improving the ability of residuals to characterize fault features. Comparing the third method with the proposed method, replacing the fixed threshold with an adaptive threshold, reduces the detection delay from 0.18 s to 0.12 s (a 33% reduction), the false alarm rate from 3.6% to 1.1% (a 69% reduction), and the missed detection rate from 2.4% to 0.8% (a 67% reduction). This shows that the adaptive threshold can dynamically adapt to varying disturbance intensities, maintaining high detection sensitivity while effectively suppressing false alarms.

The performance of the two additional comparative methods further highlights the necessity of the full three-module synergy in the proposed framework. Method 4 adopts SMO for state acquisition, which exhibits stronger robustness to parameter variations than the baseline N4SID, resulting in lower false alarm and missed detection rates than method 2. However, the absence of an adaptive decision threshold prevents it from balancing false alarm suppression and fault sensitivity under time-varying noise, leading to a 155% higher FAR and 58% longer detection delay than the proposed method.

Method 5 employs CNN-LSTM for state acquisition and integrates the proposed likelihood ratio adaptive threshold, but the lack of dedicated residual anti-interference filtering leaves the raw residual severely distorted by measurement noise. Even with the adaptive threshold mitigating some stochastic false alarms, the low-quality residual leads to a 237.5% higher missed detection rate than the proposed method, confirming that residual denoising is irreplaceable for reliable fault diagnosis in noisy scenarios.

In summary, through the three-layer progressive optimization of disturbance-aware model identification, residual anti-interference filtering, and adaptive threshold design, the proposed method achieves comprehensive improvements in fault detection speed, accuracy, and robustness.

## 5. Conclusions

This study proposed a hybrid data–mechanistic sensor fault diagnosis framework for direct-drive in-wheel motors, integrating disturbance-aware subspace identification, SVD-optimized residual anti-interference filtering, and adaptive thresholding via maximum likelihood ratio. Key findings from bench and actual vehicle experiments are summarized as follows:

(1) The predictive time-domain rolling mechanism in the N4SID algorithm effectively mitigates the impact of noise and model uncertainty, reducing current and speed identification RMSE by 56% and 61% compared to standard N4SID, respectively.

(2) The three-layer progressive optimization with disturbance-aware identification, residual filtering, and adaptive threshold achieves a 0.12 s fault detection delay, 1.1% false alarm rate, and 0.8% missed detection rate under faults, outperforming baseline methods by 33–69% in key metrics.

(3) The optimal prediction horizon balances dynamic response and disturbance suppression, validated via cross-validation.

Despite these improvements, three limitations warrant attention: (i) The method relies on continuous sensor data; severe data loss degrades identification accuracy. (ii) The SVD-based residual filter introduces about 2.5 ms computational overhead per step, posing challenges for microcontrollers with <10 MHz clock frequencies. (iii) Only gain faults were validated—the bias, drift, and intermittent faults remain untested.

While the proposed framework demonstrates robust performance for gain faults, the investigation of its efficacy against incipient faults such as bias and drift remains an immediate priority for subsequent studies. Addressing these fault types is essential to bridge the gap between the current laboratory validation and the requirements for real-world automotive applications. Future work will focus on the following aspects: (i) Integrating physics-informed neural networks with data imputation techniques to handle extreme data loss scenarios. (ii) Developing lightweight SVD algorithms via fixed-point arithmetic optimization for embedded deployment. (iii) Extending the fault set to include bias, drift, and intermittent faults, and validating the method on mass-produced vehicle platforms across diverse driving cycles.

## Figures and Tables

**Figure 1:**
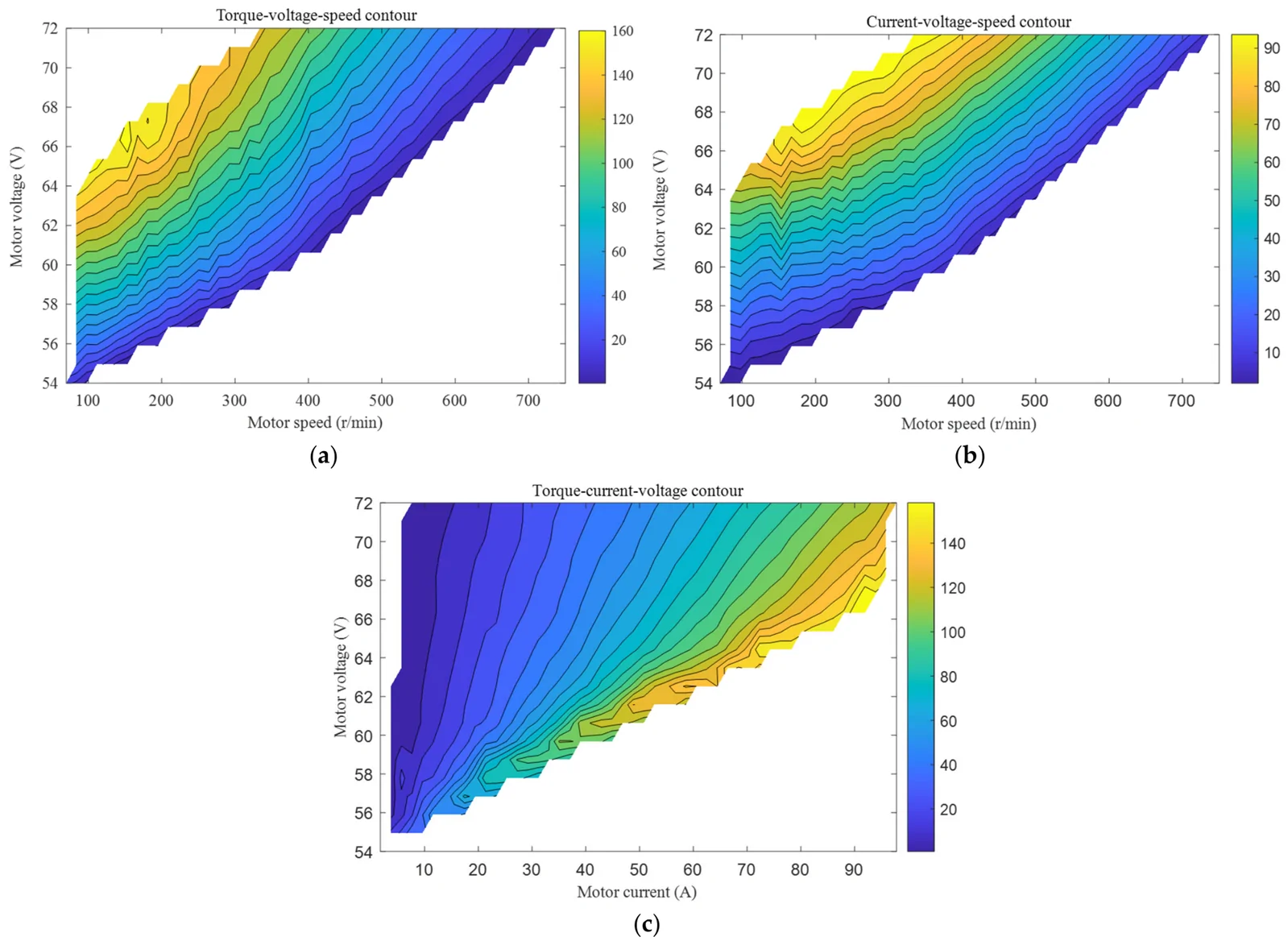
Motor state data under bench-test conditions: (**a**) torque–voltage–speed contour; (**b**) current–voltage–speed contour; (**c**) torque–current–voltage contour.

**Figure 2:**
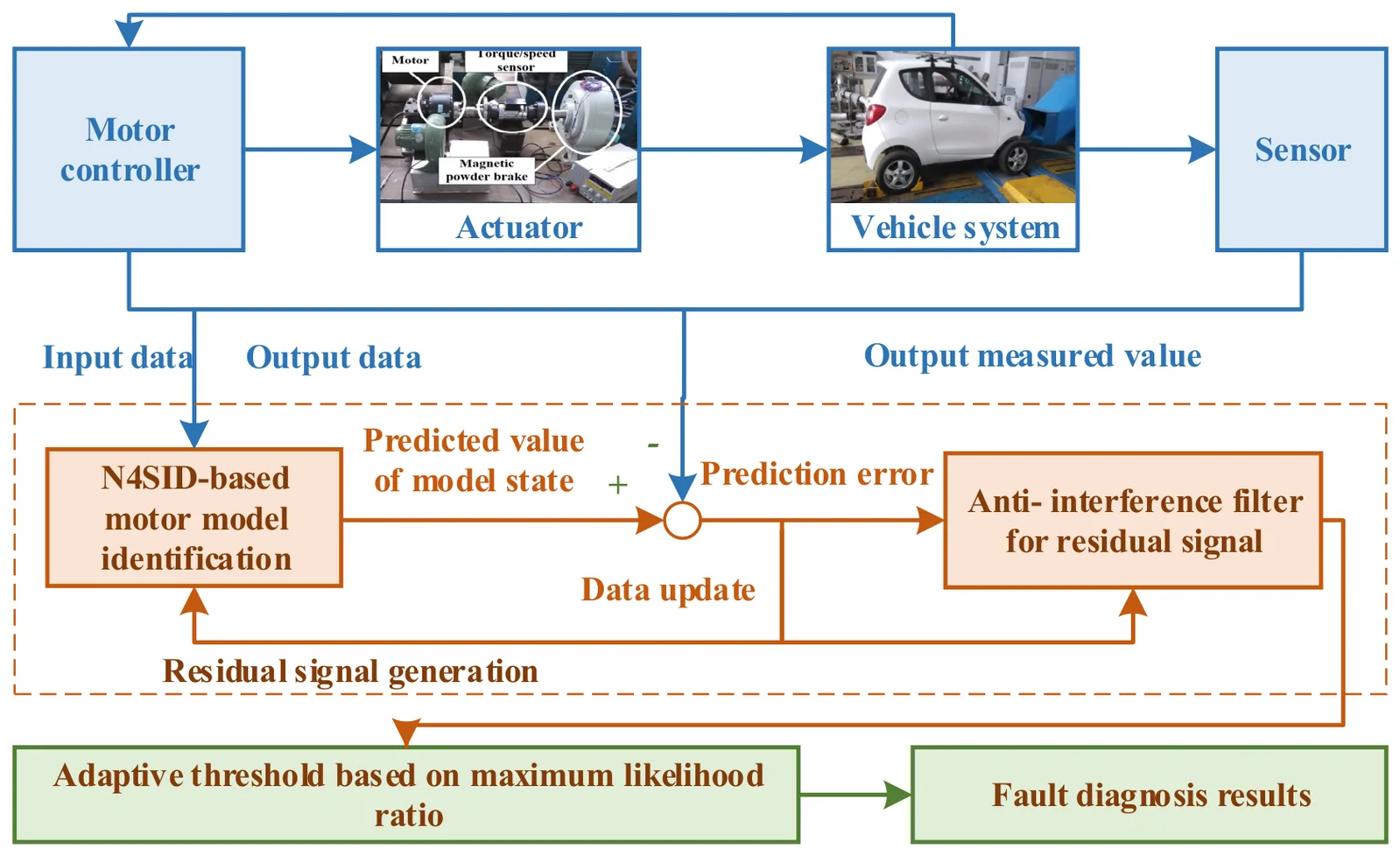
The sensor fault diagnosis scheme of the vehicle direct-drive motor system.

**Figure 3:**
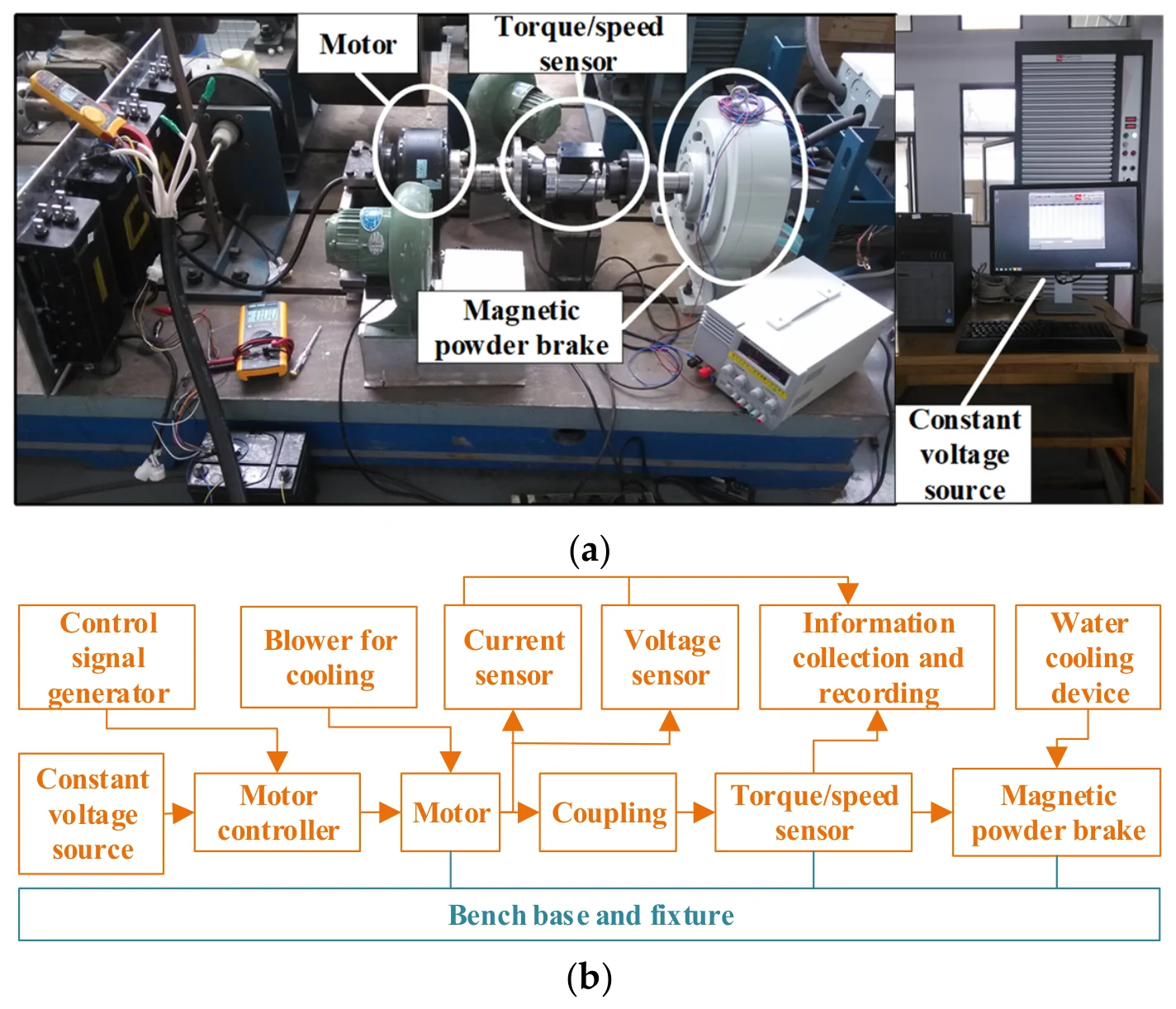
The experiment bench: (**a**) image; (**b**) schematic architecture diagram of the test bench.

**Figure 4:**
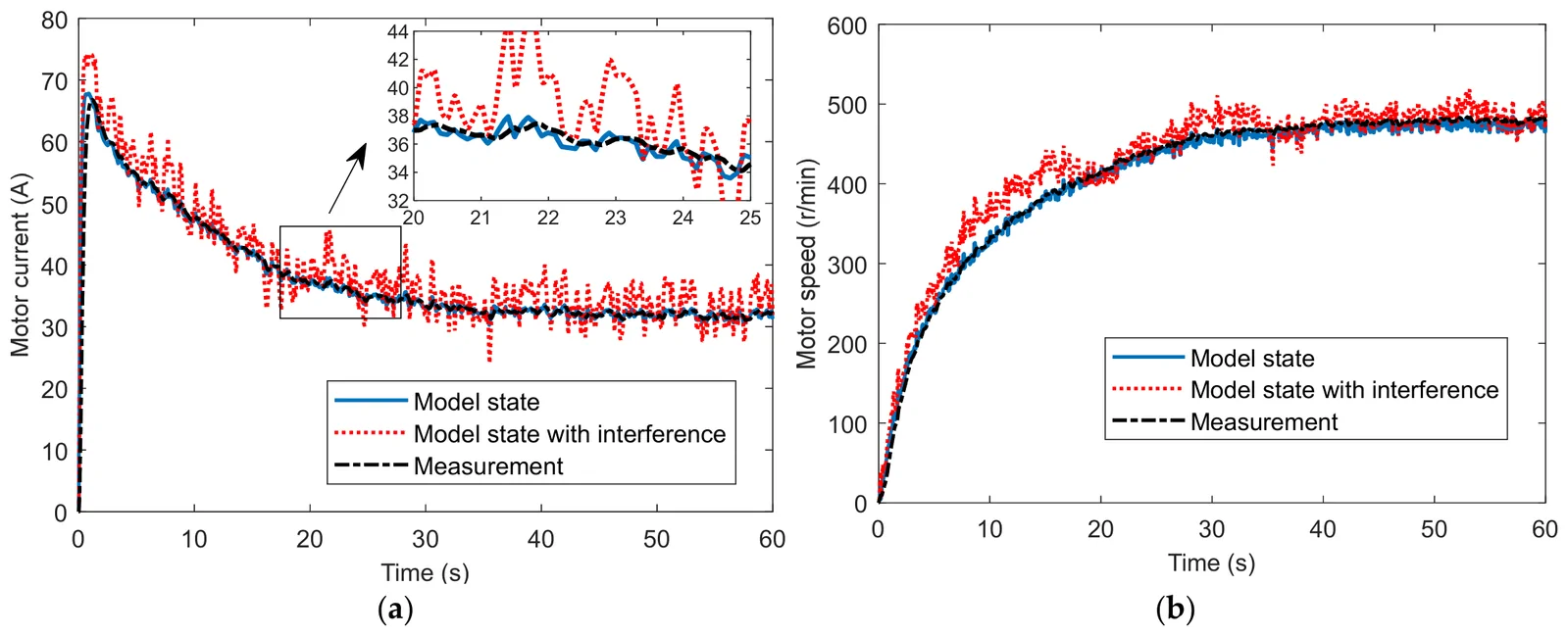
Model state identification results under the influence of noise interference: (**a**) current state value; (**b**) speed state value.

**Figure 5:**
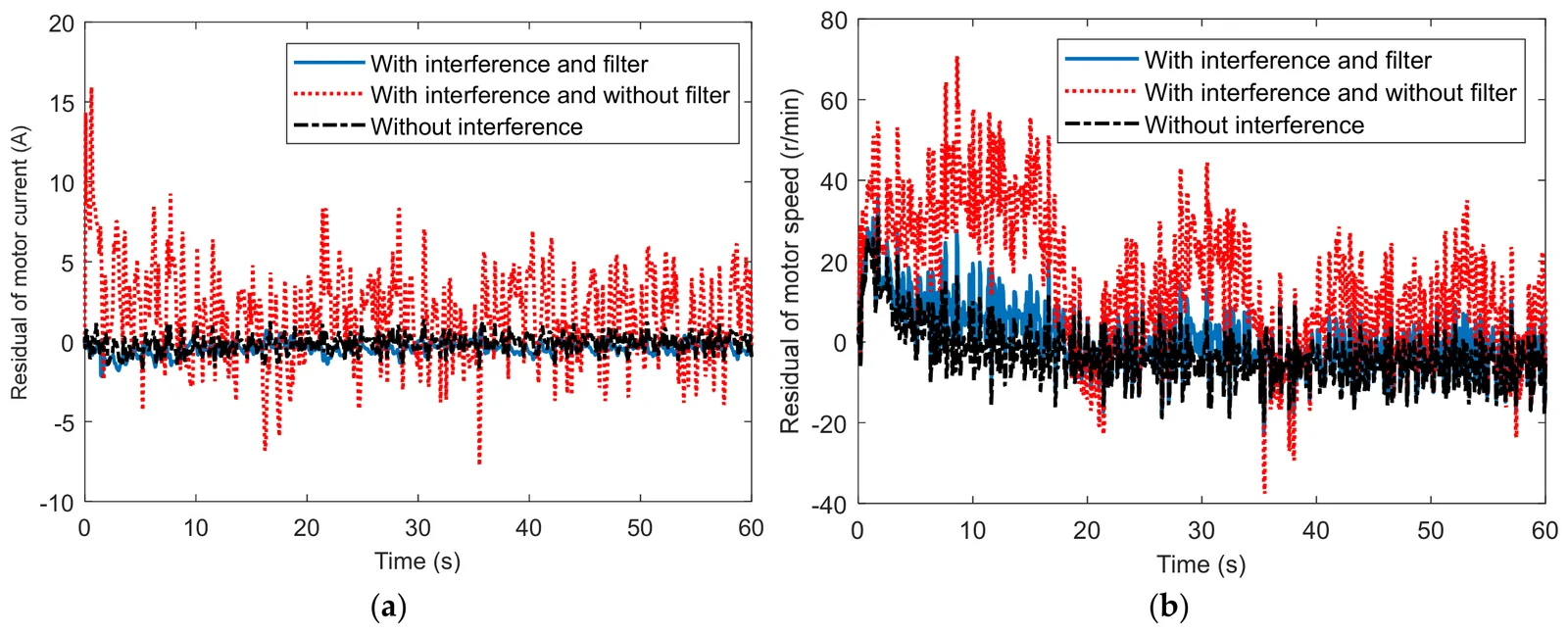
Residual filter identification results under the influence of noise interference: (**a**) current residual; (**b**) speed residual.

**Figure 6:**
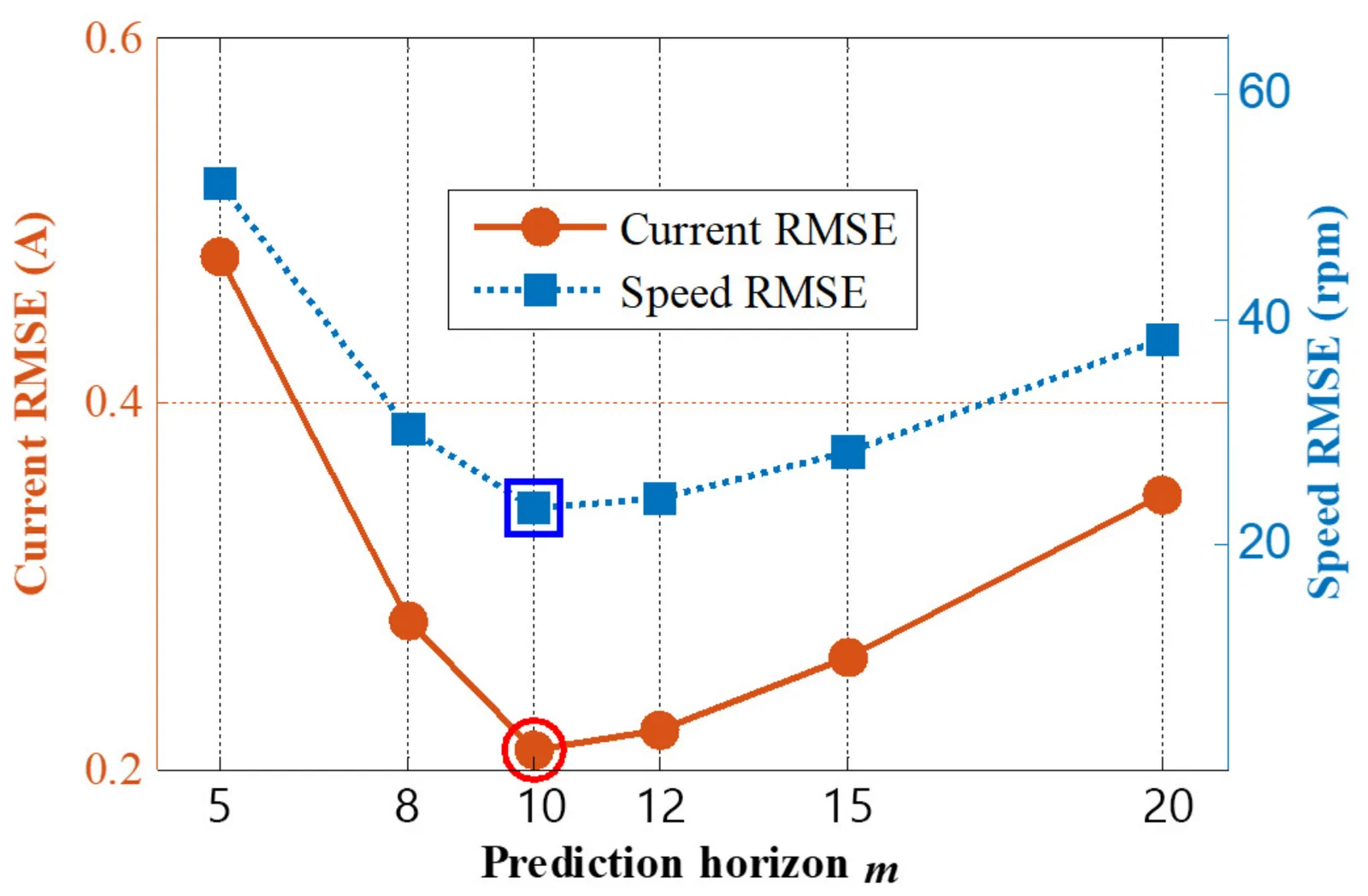
RMSE variation curves for current and speed under different prediction horizons.

**Figure 7:**
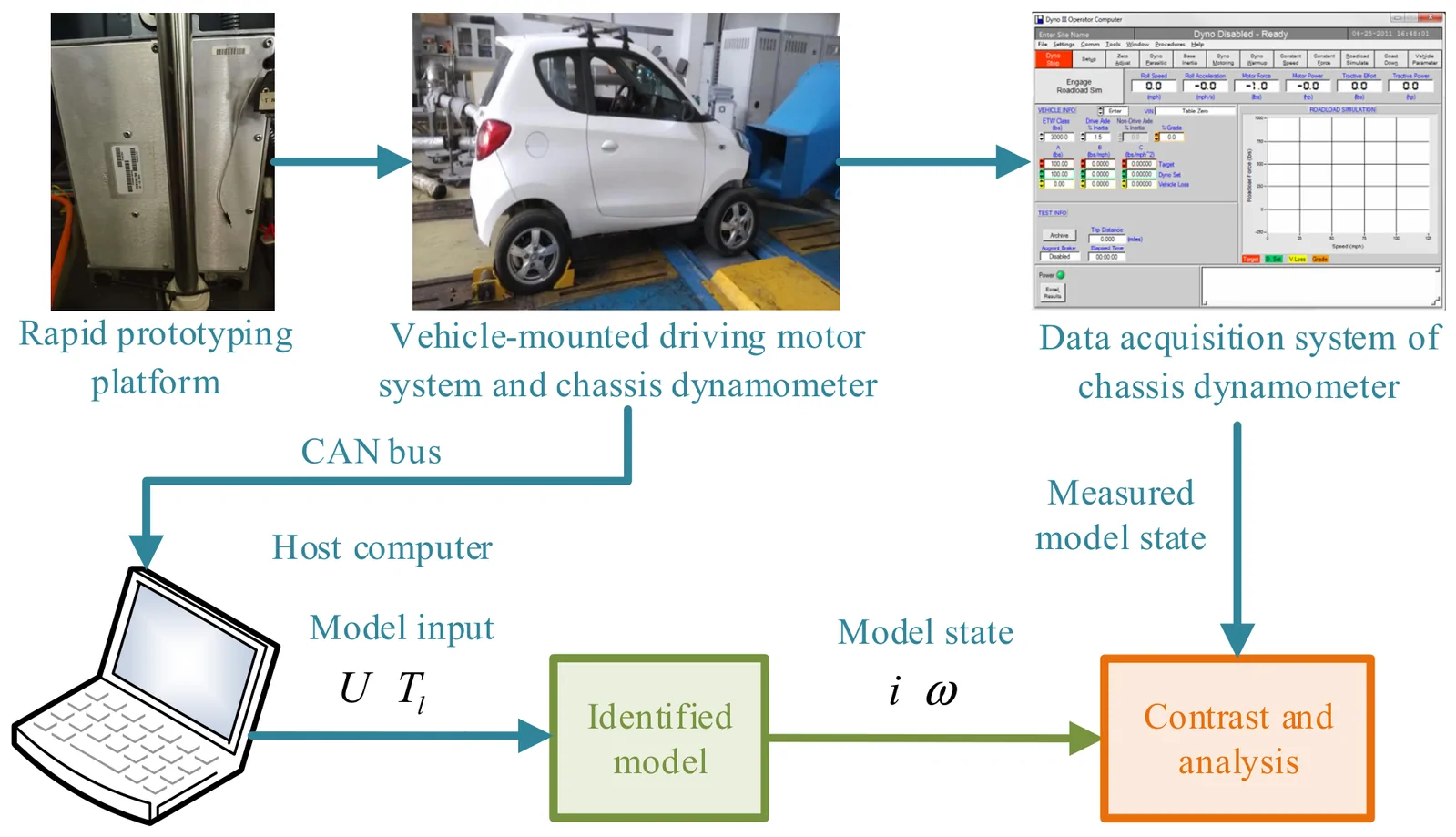
Fault diagnosis of direct-drive motor sensor based on chassis dynamometer test.

**Figure 8:**
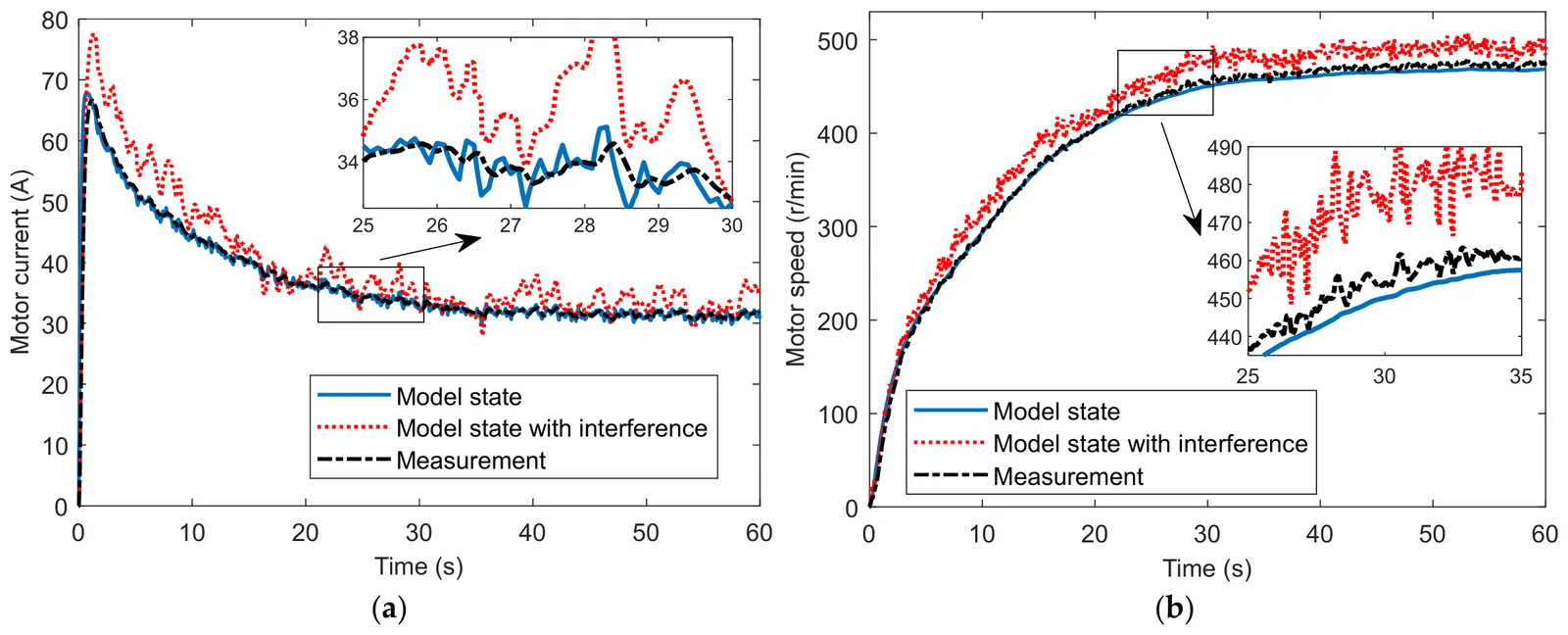
Identification results of model states under the influence of uncertainty interference: (**a**) current value; (**b**) speed value.

**Figure 9:**
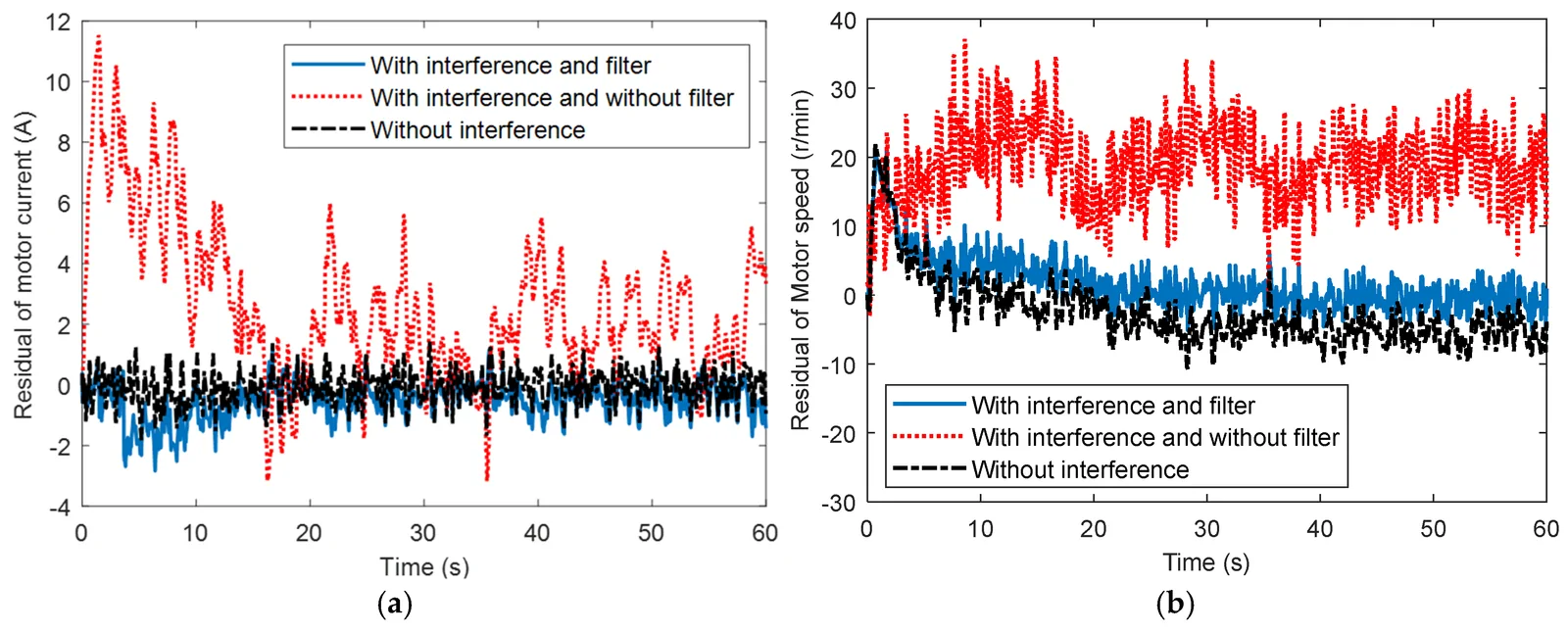
Residual filter identification results under the influence of uncertainty interference: (**a**) filtering result of current residual; (**b**) the filtering result of speed residual.

**Figure 10:**
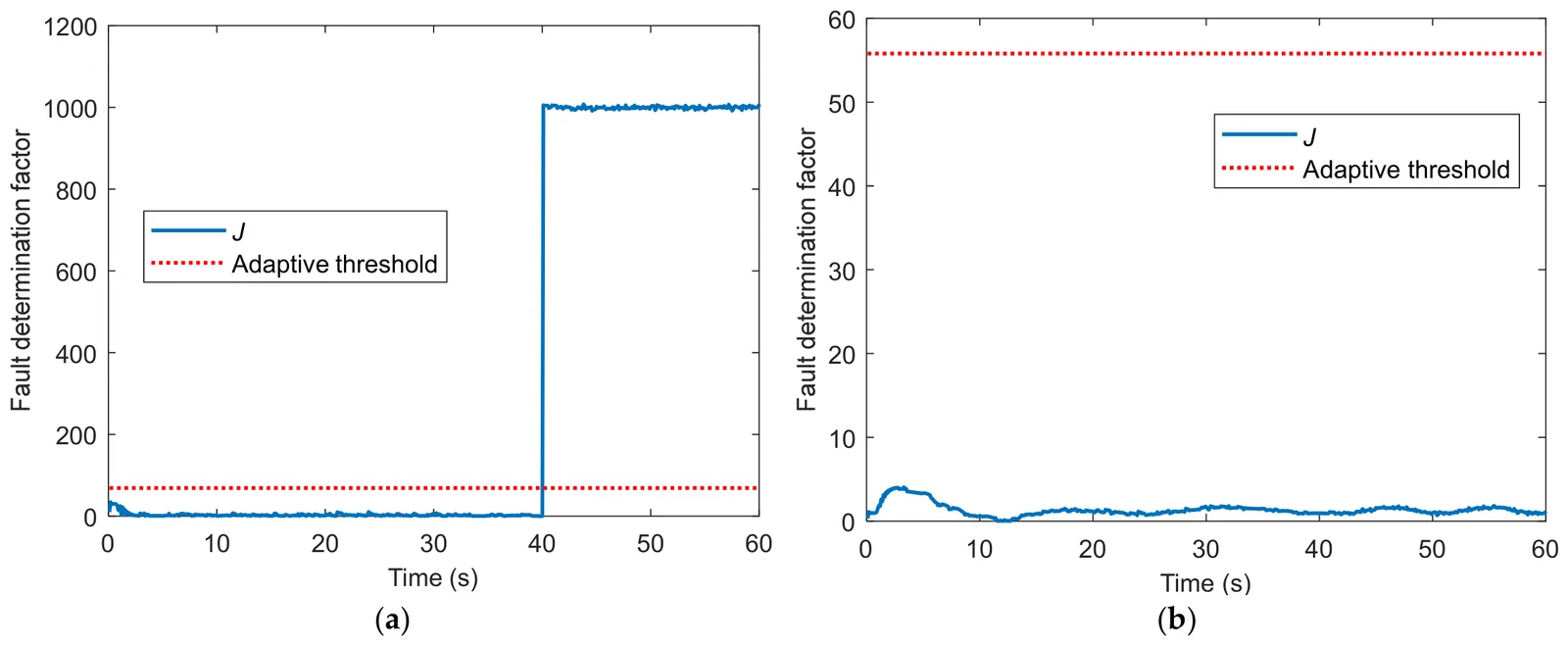
Fault diagnosis results of current sensor: (**a**) maximum likelihood ratio estimation of current residuals; (**b**) maximum likelihood ratio estimation of speed residuals.

**Figure 11:**
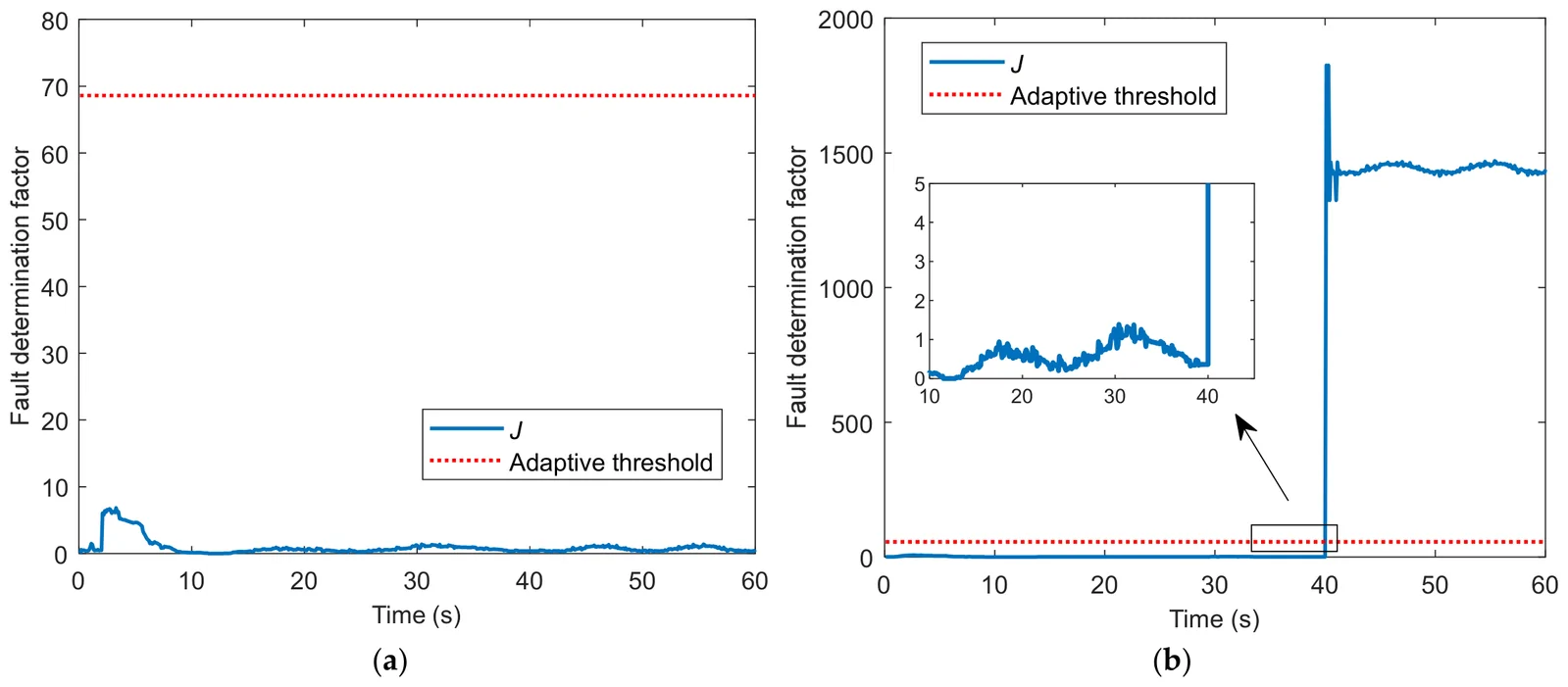
Fault diagnosis results of speed sensor: (**a**) maximum likelihood ratio estimation of current residuals; (**b**) maximum likelihood ratio estimation of speed residuals.

**Table 1:** Comparison of state identification RMSE under different SNR levels.

SNR (dB)	Current RMSE (A)	Speed RMSE (rpm)
No noise	0.08	9.2
25	0.15	16.8
20	0.21	23.0
15	0.38	41.5

**Table 2:** Statistical properties of filtered residuals under different prediction horizons.

*m*	*μ_r_* (Current)	*σ_r_* (Current)	*μ_r_* (Speed)	*σ_r_* (Speed)
5	0.018	0.042	1.56	4.83
8	0.006	0.022	0.67	2.45
10	0.002	0.015	0.21	1.78
12	0.003	0.017	0.38	2.01
15	0.009	0.028	0.95	3.42
20	0.021	0.045	1.87	5.61

**Table 3:** Comparison of fault diagnosis performance across different methods.

Method	Detection Delay (s)	FAR (%)	MDR (%)
1	0.48	12.3	10.5
2	0.35	8.2	6.5
3	0.18	3.6	2.4
4	0.19	2.8	2.2
5	0.22	3.2	2.7
Proposed	0.12	1.1	0.8

## Data Availability

The authors confirm that the data supporting the findings of this study are available within the paper.
